# Supplementary material for: Mutational signature analyses in multi-child families reveal sources of age-related increases in human germline mutations
Source: Commun Biol. 2024 Nov 6;7:1451. doi: 10.1038/s42003-024-07140-2 (PMC11541588; doi:10.1038/s42003-024-07140-2)
Supplement: Supplementary file 2 — Supplementary Information [file 42003_2024_7140_MOESM2_ESM.pdf]

Supplementary Tables and Figures

Mutational signature analyses in multi-child families suggest a key role for DNA mismatch repair in human germline *de novo* mutations

Shojaeisaadi HA[1], Schoenrock A[1,#], Meier MJ[1], Williams A[1], Norris JM[2], Palmer ND[3], Yauk CL[4], Marchetti F[1,*]

(1) Environmental Health Science and Research Bureau, Health Canada, Ottawa, ON, Canada
(2) Department of Epidemiology, Colorado School of Public Health, University of Colorado Anschutz Medical Campus, Aurora, CO, USA,
(3) Department of Biochemistry, Wake Forest School of Medicine, Winston-Salem, NC, USA,
(4) Department of Biology, University of Ottawa, Ottawa, Ontario, Canada.

[#]Present address: Research Computing Services, Carleton University, Ottawa, ON, Canada

***** Corresponding author.

Dr. Francesco Marchetti

Environmental Health Science and Research Bureau

Health Canada

251 Sir Frederick Banting Driveway,

Ottawa, ON, K1A 0K9

Canada

Email: francesco.marchetti@hc-sc.gc.ca

Tel: +1 (613) 794-1407

Submitted to Communications Biology

Supplementary Table 1. Difference in paternal age at first and last child

**IRASFS**	**Paternal Age (years)**		
**Family ID**	**First Child**	**Last Child**	**Difference**
1	22.7	28.6	5.9
2	24.5	31.2	6.7
3	24.5	38.2	13.7
4	24.9	30.4	5.5
5	17.5	20.2	2.7
6	24.4	31.3	6.9
7	16.0	21.8	5.8
8	24.1	36.8	12.7
9	16.4	22.3	5.9
10	25.0	41.2	16.2
11	26.1	40.7	14.5
12	21.1	35.9	14.8
13	24.7	34.0	9.3
**First and last child difference (yrs)**		**Average**	**9.3**
		**Median**	**6.9**
		**Minimum**	**2.7**
		**Maximum**	**16.2**

Supplementary Table 2. Cosine similarity values of reconstructed signatures by individual families

	Mutational Signatures Database		
Family	COSMIC	Germline	DNA repair KOs
IRASFS 1	0.813	**0.656**	**0.769**
IRASFS 2	0.860	**0.712**	0.805
IRASFS 3	0.904	0.810	0.805
IRASFS 4	0.906	**0.795**	0.805
IRASFS 5	0.880	0.811	**0.782**
IRASFS 6	0.886	**0.735**	**0.786**
IRASFS 7	0.867	**0.769**	0.816
IRASFS 8	0.915	**0.729**	0.828
IRASFS 9	0.878	**0.754**	**0.766**
IRASFS 10	0.938	**0.795**	0.842
IRASFS 11	0.935	0.830	0.824
IRASFS 12	0.866	0.831	0.839
IRASFS 13	0.954	0.850	0.853

Bold indicates cosine similarity values below 0.8

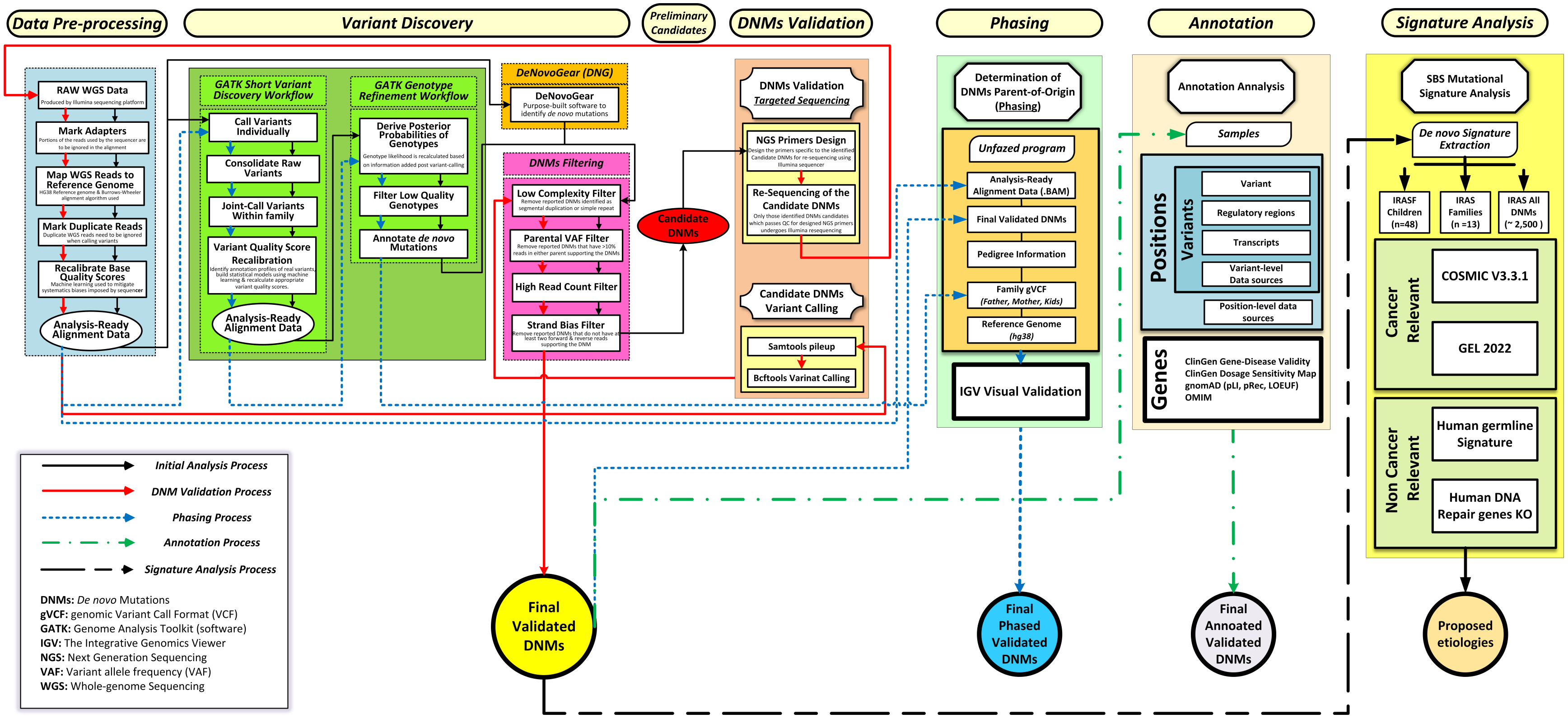

Figure S1. The bioinformatic workflow. Overall bioinformatics pipeline for SNV identification, validation and subsequent parent-of-origin phasing, annotations and mutational signature analysis.

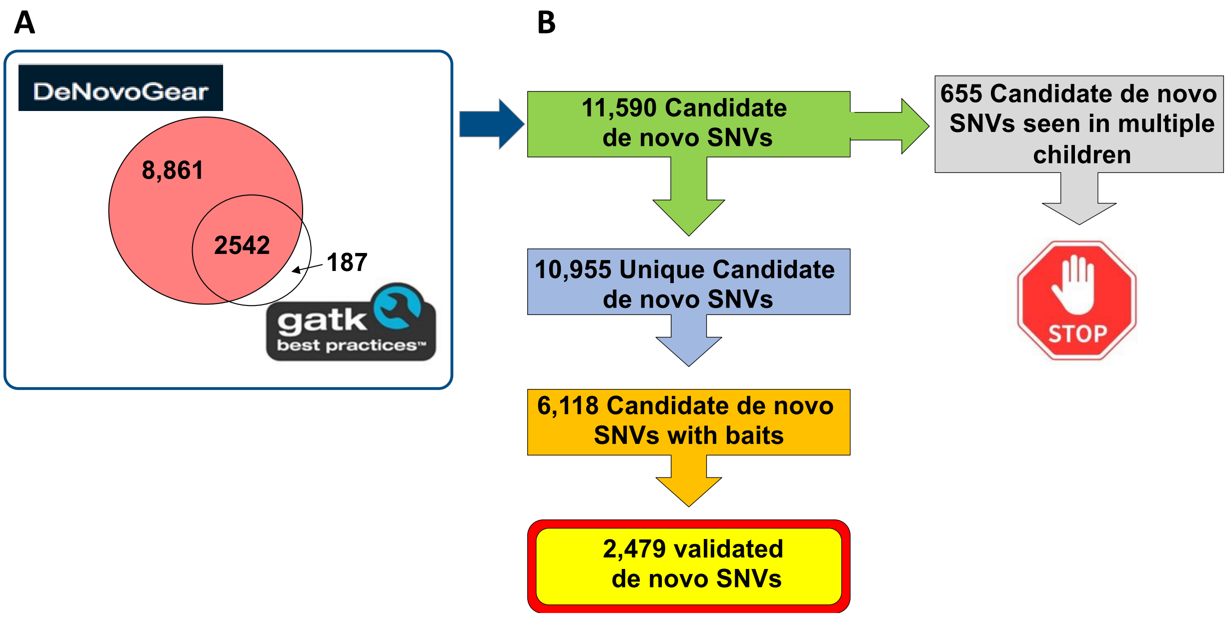

Figure S2. Process for identifying de novo single nucleotide variants (SNVs). (A) Venn diagram showing the numbers of candidate SNVs identified by DeNovoGear and GATK. **(B)** Overall, 11,590 unique SNVs were identified. The 655 candidate SNVs that were identified in several children were eliminated from further analysis generating a set of 10,955 unique candidate SNVs. Baits were successfully designed for 6,118 (~56%) candidate SNVs. Targeted resequencing of this set generated 2,479 SNVs that were successfully validated after applying the necessary filtering and QC during data processing.

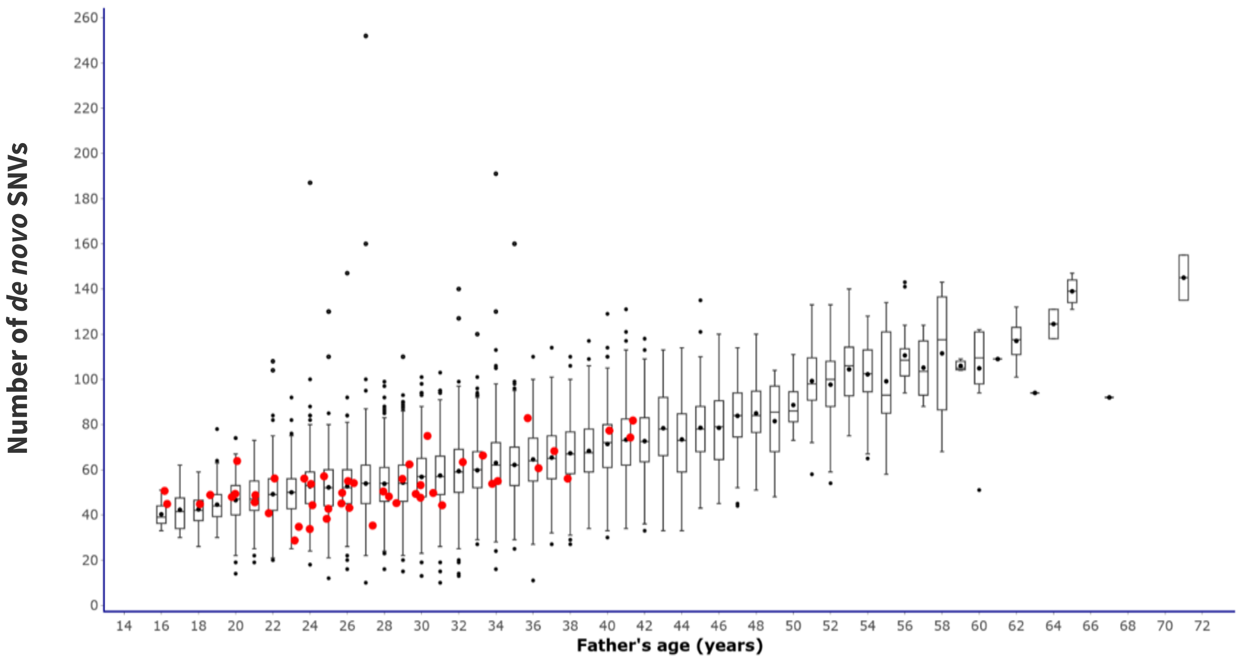

Figure S3. Comparison of *de novo* SNVs in the IRASFS cohort with other studies. Box plot distribution of the SNVs by paternal age from a dataset from 12* published trio studies with > 11,000 probands. The red dots show the number of validated SNVs for each of the 48 IRASFS probands.

* References: Michaelson *et al*., Cell 2012 Dec 21;151(7):1431-42; Kong *et al*., Nature 2012 Aug 23;488(7412):471-5; Dijk *et al*., Nat Genet. 2014 Aug;46(8):818-25; Wang et al., Cell Res. 2021 Aug;31(8):919-928; Rahbari *et al*., Nat Genet. 2016 Feb;48(2):126-133; Jónsson *et al*., Nature. 2017 Sep 28;549(7673):519-522; Kessler et al., PNAS. 2020 Feb 4;117(5):2560-2569; Halldorsson *et. al*., Science. 2019 Jan 25;363(6425):eaau1043; Sasani et al., Elife. 2019 Sep 24;8:e46922; Goldman et al., Nat Genet. 2018 Apr;50(4):487-492; Goldman et al., Nat Genet. 2016 Aug;48(8):935-9; Wilfert *et al*., Nat Genet. 2021 Aug;53(8):1125-1134.

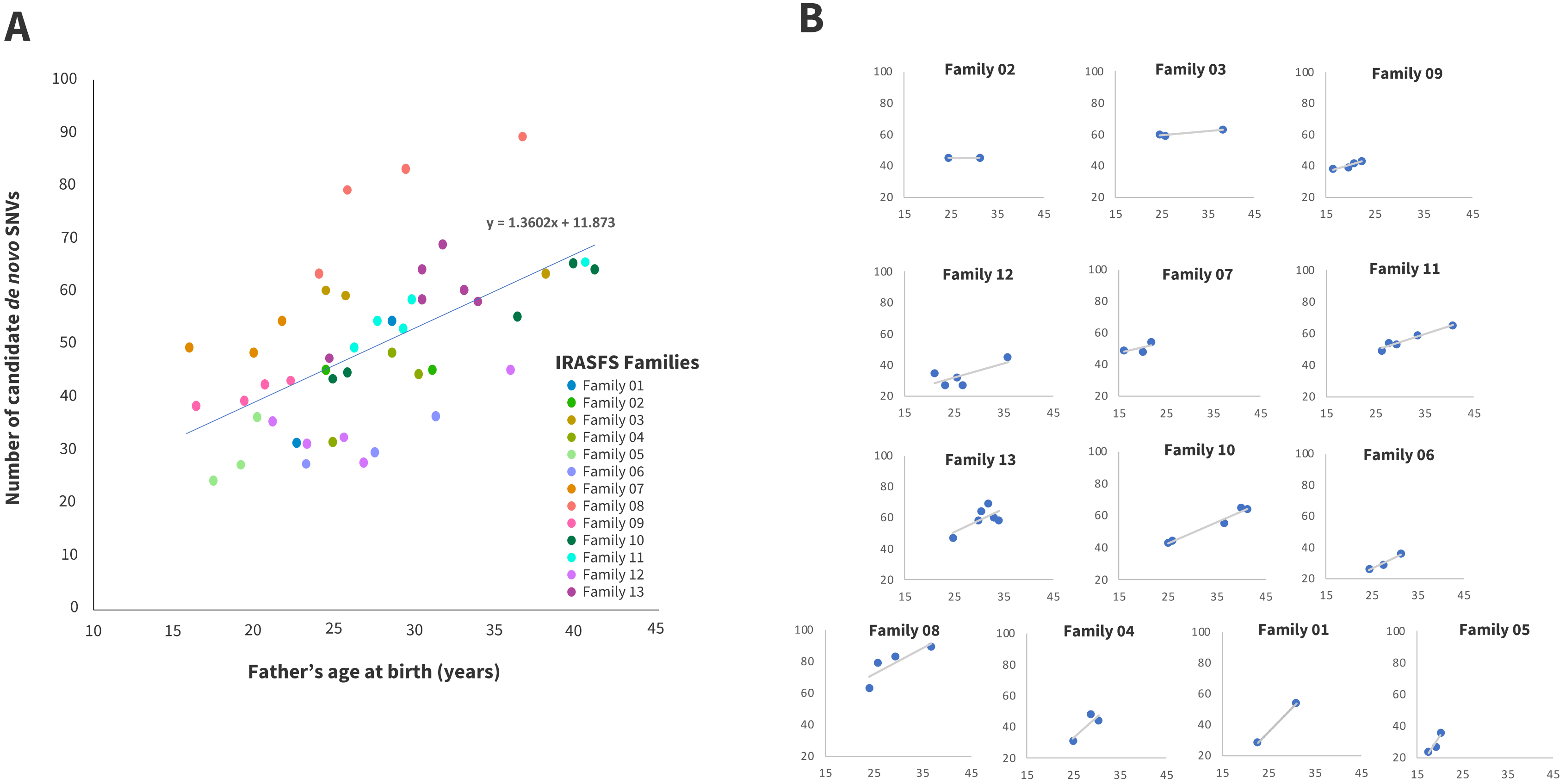

Figure S4. The distribution of candindate *de novo* SNVs identified by both GATK and DenovoGear in the IRASFS multi-child families and their correlation with paternal age. (**A**) The scatter plot represents the number of candidate *de novo* SNVs in each of the 48 children by paternal age at the time of birth. Each color represents a specific IRASFS family. The blue line represents the slope of all candidate *de novo* SNVs. (**B**) Scatter plots of the numbers of candidate *de novo* SNVs for each family relative to the father's age at each child's birth, ordered by slope from the lowest (top left corner, IRASFS Family 02) to the highest rate (bottom right corner

color, IRASFS Family 05). Regression lines and 95% confidence intervals indicate the predicted number of candidate *de novo* SNVs as a function of paternal age using a Poisson regression.

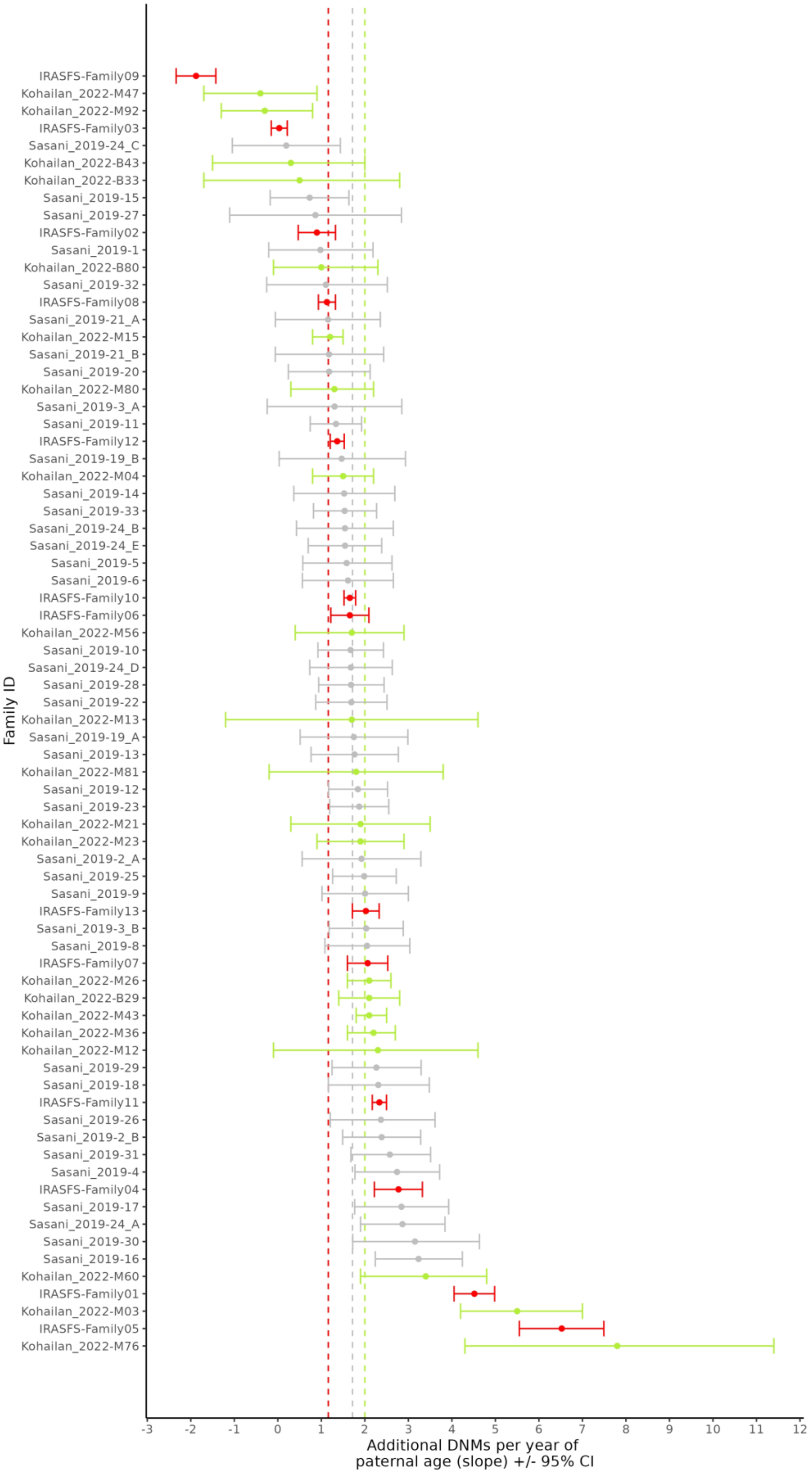
IRASFS-Family09
Kohailan_2022-M47
Kohailan_2022-M92
IRASFS-Family03
Sasani_2019-24_C
Kohailan_2022-B43
Kohailan_2022-B33
Sasani_2019-15
Sasani_2019-27
IRASFS-Family02
Sasani_2019-1
Kohailan_2022-B80
Sasani_2019-32
IRASFS-Family08
Sasani_2019-21_A
Kohailan_2022-M15
Sasani_2019-21_B
Sasani_2019-20
Kohailan_2022-M80
Sasani_2019-3_A
Sasani_2019-11
IRASFS-Family12
Sasani_2019-19_B
Kohailan_2022-M04
Sasani_2019-14
Sasani_2019-33
Sasani_2019-24_B
Sasani_2019-24_E
Sasani_2019-5
Sasani_2019-6
IRASFS-Family10
IRASFS-Family06
Kohailan_2022-M56
Sasani_2019-10
Sasani_2019-24_D
Sasani_2019-28
Sasani_2019-22
Kohailan_2022-M13
Sasani_2019-19_A
Sasani_2019-13
Kohailan_2022-M81
Sasani_2019-12
Sasani_2019-23
Kohailan_2022-M21
Kohailan_2022-M23
Sasani_2019-2_A
Sasani_2019-25
Sasani_2019-9
IRASFS-Family13
Sasani_2019-3_B
Sasani_2019-8
IRASFS-Family07
Kohailan_2022-M26
Kohailan_2022-B29
Kohailan_2022-M43
Kohailan_2022-M36
Kohailan_2022-M12
Sasani_2019-29
Sasani_2019-18
IRASFS-Family11
Sasani_2019-26
Sasani_2019-2_B
Sasani_2019-31
Sasani_2019-4
IRASFS-Family04
Sasani_2019-17
Sasani_2019-24_A
Sasani_2019-30
Sasani_2019-16
Kohailan_2022-M60
IRASFS-Family01
Kohailan_2022-M03
IRASFS-Family05
Kohailan_2022-M76
Family ID
-3
-2
-1
0
1
2
3
4
5
6
7
8
9
10
11
12
Additional DNMs per year of
paternal age (slope) +/- 95% CI

72

Figure S5. Comparison of paternal age effects among multi-sibling families from diverse ethnic backgrounds. Slope ± 95% confidence interval (CI) of the IRASFS cohort (red) compared with the CEPH/Utah multi-sibling families (Sasani *et al*., 2019; gray) and Middle-East multiplex families (Kohailan *et al*., 2022; green). Each family is sorted in order of increasing slope. Dashed vertical lines indicate the combined paternal age effect based on all families within a study, with colours representing the corresponding cohorts: 1.29 de novo SNVs/year, 95% CI: 1.44-1.57, $p < 0.0001$ for IRASFS (red); 1.72 de novo SNVs/year, 95% CI: 1.58–1.85, $p < 2e\text{-}16$ for the CEPH/Utah (grey); and, 1.36 de novo SNVs/year, 95% CI: 1.11–1.61, $p = 1 \times 10^{-22}$ for Middle-East multiplex families cohort (green).
