# Supplementary material for: Mutational signature analyses in multi-child families reveal sources of age-related increases in human germline mutations
Source: Commun Biol. 2024 Nov 6;7:1451. doi: 10.1038/s42003-024-07140-2 (PMC11541588; doi:10.1038/s42003-024-07140-2)
Supplement: Supplementary file 3 — Description of Additional Supplementary Materials [file 42003_2024_7140_MOESM3_ESM.pdf]

Description of Additional Supplementary Files

File name: Supplementary Data 1

Description: The file contains nine tabs. The titles and description of each tab are: Figure 2: Complete list of validated SNVs by proband. Figure 3: Validated SNVs with intercept and slope data Figure 4: Validated SNVs by parent of origin Figure 5A: 96-trinucleotide spectrum for SNVs identified in all children from this study Figure 5B: 96-trinucleotid spectrum for SNVs from children born from the youngest fathers (33.1 years of age) Figure 6A: Percentages of each of the 96 trinucleotides for signature reconstruction using the COSMIC database Figure 6B: Percentages of each of the 96 trinucleotides for signature reconstruction using the Germline database Figure 6C: Percentages of each of the 96 trinucleotides for signature reconstruction using the DNA repair KO database
