# Supplementary material for: Mutational signature analyses in multi-child families reveal sources of age-related increases in human germline mutations
Source: Commun Biol. 2024 Nov 6;7:1451. doi: 10.1038/s42003-024-07140-2 (PMC11541588; doi:10.1038/s42003-024-07140-2)
Supplement: Supplementary file 5 — Reporting summary [file 42003_2024_7140_MOESM5_ESM.pdf]

nature portfolio

Corresponding author(s): Francesco Marchetti

Last updated by author(s): Jul 20, 2024

Reporting Summary

Nature Portfolio wishes to improve the reproducibility of the work that we publish. This form provides structure for consistency and transparency in reporting. For further information on Nature Portfolio policies, see our Editorial Policies and the Editorial Policy Checklist.

Statistics

For all statistical analyses, confirm that the following items are present in the figure legend, table legend, main text, or Methods section.

n/a	Confirmed	
☐	☒	The exact sample size (*n*) for each experimental group/condition, given as a discrete number and unit of measurement
☐	☒	A statement on whether measurements were taken from distinct samples or whether the same sample was measured repeatedly
☐	☒	The statistical test(s) used AND whether they are one- or two-sided<br>*Only common tests should be described solely by name; describe more complex techniques in the Methods section.*
☒	☐	A description of all covariates tested
☐	☒	A description of any assumptions or corrections, such as tests of normality and adjustment for multiple comparisons
☐	☒	A full description of the statistical parameters including central tendency (e.g. means) or other basic estimates (e.g. regression coefficient) AND variation (e.g. standard deviation) or associated estimates of uncertainty (e.g. confidence intervals)
☐	☒	For null hypothesis testing, the test statistic (e.g. *F*, *t*, *r*) with confidence intervals, effect sizes, degrees of freedom and *P* value noted<br>*Give P values as exact values whenever suitable.*
☒	☐	For Bayesian analysis, information on the choice of priors and Markov chain Monte Carlo settings
☒	☐	For hierarchical and complex designs, identification of the appropriate level for tests and full reporting of outcomes
☐	☒	Estimates of effect sizes (e.g. Cohen's *d*, Pearson's *r*), indicating how they were calculated

Our web collection on statistics for biologists contains articles on many of the points above.

Software and code

Policy information about availability of computer code

Data collection	None
Data analysis	The workflow used to do signature analyses is available on github (https://github.com/hashoja/SNVs_DNMs_IRASFS); publicly available open source R libraries and Signature analysis tools (e.g. SigProfiler Tools)

For manuscripts utilizing custom algorithms or software that are central to the research but not yet described in published literature, software must be made available to editors and reviewers. We strongly encourage code deposition in a community repository (e.g. GitHub). See the Nature Portfolio guidelines for submitting code & software for further information.

Data

Policy information about availability of data

All manuscripts must include a data availability statement. This statement should provide the following information, where applicable:

- Accession codes, unique identifiers, or web links for publicly available datasets
- A description of any restrictions on data availability
- For clinical datasets or third party data, please ensure that the statement adheres to our policy

Whole genome sequencing data from the IRASFS cohort is available under dbGaP Study Accession: php001008.v1.p1 and by contacting Dr. Nichole Palmer at nallred@wakehealth.edu. All 2,479 validated SNVs are listed in Supplementary Data. All other data are available from the corresponding author on reasonable request

Research involving human participants, their data, or biological material

Policy information about studies with human participants or human data. See also policy information about sex, gender (identity/presentation), and sexual orientation and race, ethnicity and racism.

Reporting on sex and gender	Our human samples include both male and female (sex-based). It was collected during data sampling as well as through Bioinformatics data analysis.
Reporting on race, ethnicity, or other socially relevant groupings	The IRASFS cohort focus on individuals with Mexican-American ethnicity. We have added a paragraph in the material and methods providing details of the IRASFS cohort and relevant references.
Population characteristics	On average, selected families had ~4 children (mean ± SD: 3.7 ± 1.2) with a mean paternal age of 27.5 ± 6.4 years at the time of birth with a minimum and maximum age of 16.4 and 41.2 years old, respectively. Average paternal age difference between the first and last child was 9.3 ± 4.9 years.
Recruitment	The recruitment for human IRASFS cohort has been described in its original paper : Ann Epidemiol. 2003 Apr;13(4):211-7. doi: 10.1016/s1047-2797(02)00412-x.
Ethics oversight	The use and handling of human samples in this study was approved by the Research Ethic Board of Health Canada and Public Health Agency of Canada under protocol REB 2016-001H. For the IRASFS cohort, all study protocols were approved by the Institutional Review Board of each participating clinical and analysis site and all participants provided written informed consent.

Note that full information on the approval of the study protocol must also be provided in the manuscript.

Field-specific reporting

Please select the one below that is the best fit for your research. If you are not sure, read the appropriate sections before making your selection.

☒ Life sciences ☐ Behavioural & social sciences ☐ Ecological, evolutionary & environmental sciences

For a reference copy of the document with all sections, see nature.com/documents/nr-reporting-summary-flat.pdf

Life sciences study design

All studies must disclose on these points even when the disclosure is negative.

Sample size	Number of mutations are reported for all 48 probands presented in the study
Data exclusions	SNVs were excluded based on our QC and filtering criteria (e.g. parental alternate allele fraction (AAF) > 10%)
Replication	No
Randomization	No
Blinding	No, but all analyses were conducted by automated pipelines.

Reporting for specific materials, systems and methods

We require information from authors about some types of materials, experimental systems and methods used in many studies. Here, indicate whether each material, system or method listed is relevant to your study. If you are not sure if a list item applies to your research, read the appropriate section before selecting a response.

Materials & experimental systems

n/a	Involved in the study	
☒	☐	Antibodies
☒	☐	Eukaryotic cell lines
☒	☐	Palaeontology and archaeology
☒	☐	Animals and other organisms
☒	☐	Clinical data
☒	☐	Dual use research of concern
☒	☐	Plants

Methods

n/a	Involved in the study	
☒	☐	ChIP-seq
☒	☐	Flow cytometry
☒	☐	MRI-based neuroimaging

Plants

Seed stocks

Report on the source of all seed stocks or other plant material used. If applicable, state the seed stock centre and catalogue number. If plant specimens were collected from the field, describe the collection location, date and sampling procedures.

Novel plant genotypes

Describe the methods by which all novel plant genotypes were produced. This includes those generated by transgenic approaches, gene editing, chemical/radiation-based mutagenesis and hybridization. For transgenic lines, describe the transformation method, the number of independent lines analyzed and the generation upon which experiments were performed. For gene-edited lines, describe the editor used, the endogenous sequence targeted for editing, the targeting guide RNA sequence (if applicable) and how the editor was applied.

Authentication

Describe any authentication procedures for each seed stock used or novel genotype generated. Describe any experiments used to assess the effect of a mutation and, where applicable, how potential secondary effects (e.g. second site T-DNA insertions, mosiacism, off-target gene editing) were examined.
